# Apex and ApeTouch: Development of a Portable Touchscreen System and Software for Primates at Zoos

**DOI:** 10.3390/ani12131660

**Published:** 2022-06-28

**Authors:** Christopher Flynn Martin, Akiho Muramatsu, Tetsuro Matsuzawa

**Affiliations:** 1 Indianapolis Zoological Society, Indianapolis, IN 46222, USA; 2 Department of Informatics, Indiana University, Bloomington, IN 47405, USA; 3 Kyoto University Institute for Advanced Study, Kyoto University, Kyoto 606-8501, Japan; akiho.muramatsu@gmail.com; 4 Department of Pedagogy, Chubu Gakuin University, Gifu 504-8037, Japan; tetsuro.matsuzawa@gmail.com; 5 Division of Humanities and Social Sciences, California Institute of Technology, Pasadena, CA 91125, USA

**Keywords:** touchscreen, primate cognition, primate software, enrichment, welfare, zoo, animal–computer interaction

## Abstract

**Simple Summary:**

Zoos are increasingly looking at technology-based enrichment as a way to improve the welfare of primates in their care. Touchscreen tasks are an option that have a long and established history of usage by primates in research settings as well as a history in zoos. However, the barrier-to-entry is high for new zoos interested in adopting the primate touchscreen method. There are no pre-built and zoo-specific hardware and software options available, so zoos must assemble touchscreen systems on their own and write their own software or use pre-existing research-based software that is not ideally suited to zoo settings and applications. To remedy this, we developed a pre-built portable touchscreen system named Apex along with easy-to-operate primate software named ApeTouch; both are available for zoos to acquire. Our system and software offer enrichment, research, and husbandry applications. To illustrate the utility of these tools, we report on a training study with four species of zoo-housed macaques using the Apex machine and ApeTouch software.

**Abstract:**

We report on the development and testing of a portable touchscreen apparatus and accompanying software program for primate enrichment, cognitive research, and husbandry applications. For zoos considering using technology to bolster scientific efforts or enhance the welfare of primates in their care, touchscreen activities offer a solution that has a long and proven record of primate use in laboratory settings as well as a history of usage in the zoo world. We review the options that are available for zoos to build their own touchscreen systems and we offer as an alternative our pre-built apparatus, Apex, and primate software suite, ApeTouch, both of which are tailored for use in a zoo setting. The efficacy and utility of these tools are demonstrated in a training study with four macaque groups of different species that were previously naïve to touchscreens. All of the groups in the study learned to use the device and displayed a consistent engagement with the touchscreen tasks over 95 daily sessions of exposure. In the final stage of the training, two of the four groups displayed an above-chance level performance on a numerical sequencing task.

## 1. Introduction

Primates in their natural habitats are constantly solving problems. They must navigate across vast and dynamic environments, efficiently forage for food, and negotiate their way through complex social relationships and hierarchies. Overcoming these challenges requires a repertoire of mental and physical skills and abilities. In zoo settings, many of these challenges are reduced and reliance on species-typical behavior and cognition is correspondingly diminished. As a potential remedy, there is growing interest among zoos to leverage modern technology to create dynamic problem-solving opportunities for primates. In particular, computer touchscreen tasks for primates, which have traditionally been the province of research laboratories, are making headway into zoos as a means of enriching the lives of animals, contributing to scientific discoveries, aiding in husbandry practices, and enhancing visitor experiences [1].

Zoos are increasingly becoming primate research hubs [2,3] and computer touchscreen methods are a prevalent method at zoos for studying primate cognition [4]. Touchscreens are intuitive for primates to understand because of the one-to-one mapping of stimuli presentation and the touch response on the screen [5]. They offer a method that is precise, objective, and capable of detailed record-keeping [6,7]. Frequent and even daily usage of computer touchscreens by great apes, often in front of zoo guests, began at the Smithsonian National Zoo Orangutan ThinkTank [8] and has since become a routine activity for great apes at Lincoln Park Zoo [9], Kyoto City Zoo [10], Zoo Atlanta [11], and Indianapolis Zoo [12]. Touchscreen tasks have also been used consistently, but less frequently, with great apes at Leipzig Zoo [13], Edinburgh Zoo [14], Basel Zoo [15], and Detroit Zoo [16]. These cases often involve zoo–academia collaborations [17]; the methods typically include tasks and equipment formerly developed and refined in university laboratory settings to produce publishable findings on primate cognition. Due to the zoo setting, several of these efforts have also incorporated the additional considerations of enhancing animal welfare [18] and educational outreach for zoo visitors [12,19].

In parallel to research efforts, computerized tasks for primates at zoos are also gaining popularity as a form of “cognitive enrichment” [20,21] and “digital enrichment” [22]. Automated electronic devices used solely for primate enrichment purposes have a history going back to Markowitz [23], who used tasks involving automated lights, buttons, and food dispensers to incentivize locomotor activity in zoo-housed gibbons and mandrills. More recently, the growing field of animal–computer interaction (ACI), which emphasizes iterative user-centric design processes and consent on the part of the animal [24], has spawned a variety of projects that use electronics as an enrichment for primates, often without the practice of provisioning food rewards. Efforts along these lines include systems involving projection screens and sensors at Kyoto City Zoo [25] and Melbourne Zoo [26] to create immersive interactive activities for great apes, and touchscreen-based musical, video, and puzzle apps for orangutans at Zoo Atlanta on a screen embedded in a large fake tree in their enclosure [27]. Giving primates choice and control over environmental factors has also been investigated in a study with white-faced sakis using proximity sensors to activate different sounds and videos in their enclosure [28]. On the one hand, these approaches represent significant advances in technology-based enrichment and animal–computer interaction methods, but on the other hand, the lack of food reinforcement often leads to a rapid decline in usage rates following the initial exposure of the primates to the devices [29].

In addition to research and enrichment, computer tasks have also had primate care and husbandry applications in both laboratory and zoo settings. Assessments of primate preferences for different food items, which has direct benefits on determining feeding practices, have been undertaken with macaques, gorillas, and chimpanzees at Lincoln Park Zoo [30] and with chimpanzees at Kyoto University [31]. Likewise, a touchscreen-based assessment of audio preferences has been undertaken with gorillas at Detroit Zoo [32] and with orangutans at Toronto Zoo [33]. These studies can inform zoo staff and exhibit designers about the potential welfare costs and benefits of background noise in primate living spaces. Noise effects on primate welfare have also been examined in the context of so-called “cognitive bias” tasks, which aim to evaluate mood and emotions in animals (see [34] for a review). Using a cognitive bias paradigm, Cronin and colleagues at Lincoln Park Zoo showed a slowing of primate responses to touchscreen tasks caused by anthropogenic noises in their environment [35]. Touchscreen studies conducted by Vonk and colleagues (Detroit Zoo), Hopper and colleagues (Lincoln Park Zoo), and Allritz and colleagues (Leipzig Zoo) have further examined the emotional moods of animals with other cognitive bias methods, including emotional Stroop tests [13,16,36] and ambiguous cue tests [37]. In another study on the welfare effects of touchscreen studies, Leeds and Lukas at Cleveland Metroparks Zoo found that routine cognitive testing increased affiliative behaviors between mandrills subsequent to touchscreen testing sessions [38]. Touchscreen tasks have also been an effective means of promoting physical rehabilitation in great apes. At Kyoto University, a 24-year-old male chimpanzee named Reo developed acute transverse myelitis resulting in impaired leg movements [39]. To encourage locomotion during his recovery, a touchscreen was installed on one wall of his room connected to a universal feeder machine two meters away on the opposite wall. The automated setup led to a summative travel distance between the touchscreen and feeder that averaged 506 m a day compared with the baseline mean of 137 m without the automated task turned on [40].

For zoos interested in adopting touchscreen activities for their primates, there are a variety of apparatuses described in the scientific literature that combine a touchscreen, computer, automated feeding machine, enclosure box, and task software. These include wall-embedded stations [12,13,41,42,43], standalone kiosks [44], cart-based systems [45,46,47], systems designed for standardized macaque cage windows [48,49,50,51], and portable cage-mounted apparatus [11,52,53]. With the exception of the Lafayette Instruments Intellistation [53], all of these devices require potential users to procure, assemble, and fabricate a variety of electronic and material components, albeit in a few cases with detailed guidance from published writings [48,52]. On the software side, these apparatuses are all built for use with research-based software platforms that mostly lack an enrichment or husbandry focus and they often require expertise to operate, from substantial programming experience (e.g., custom applications, PsychoPy, MonkeyLogic, MWorks) to skills required for setting up trial sequence and stimuli sets (E-Prime, Presentation).

The aforementioned apparatuses are valuable tools for the scientific contexts for which they were designed to be used, but the singular focus on research tasks and the technical know-how required to build and operate them are likely to be prohibitive factors for many zoos interested in adopting primate touchscreens. Rather, many zoos may benefit more from a pre-built commercially available apparatus and easy-to-operate software that includes not only research tasks, but also dedicated training apps, enrichment games, and husbandry aides. Moreover, such a machine should be easily installed, operated, and maintained by keeper staff without necessarily needing the involvement or assistance of specialized fabricators, programmers, or outside researchers. Our solution to fill this void is a portable touchscreen system named Apex and a software suite named ApeTouch. These tools are currently in use at several primate facilities, including zoos, and are manufactured and distributed by Zenrichment (a company operated by C Martin, the first author of this paper).

Descriptions of the Apex machine and ApeTouch software are provided in the sections below as well as a study illustrating their use with four macaque species at the Japan Monkey Centre (JMC Zoo) in Inuyama, Japan. The JMC Zoo macaques in our study had little to no prior experience with computer tasks. Thus, the primary goal of the study was to develop and test, using the Apex machine and ApeTouch software, a robust training methodology for touchscreen-inexperienced primates.

## 2. Materials and Methods

### 2.1. Apex Apparatus

**Figure 1:**
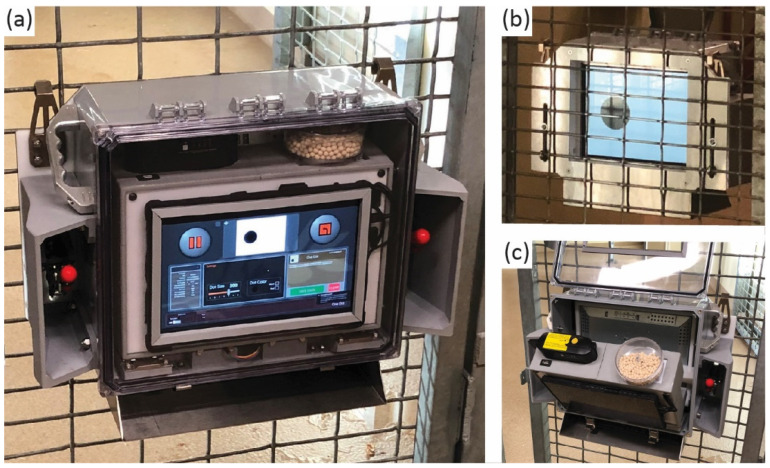
(**a**) View of the Apex machine from behind where the human operator is situated. (**b**) View of the front, where the primate is situated. (**c**) View of the Apex machine with the back cover open and the core box extended outward.

Apex is a portable touchscreen system that was developed to be compact, wireless (battery-powered), and ape-proof (Figure 1). The key features of Apex that make it ideal for use at zoos are portability and usability. The machine is wireless (no power cables) and weighs 30 pounds, so it can easily be moved between rooms and enclosures with cage mesh walls. It can also be attached and detached from caging whilst primates are present on the other side of the mesh. To enhance the usability, a secondary human-facing touchscreen allows for staff to navigate and control the software without having to interface directly with the primate-facing touchscreen. The machine is capable of set-it-and-forget-it functionality, so primates can proceed through multiple tasks for extended periods of time without a staff member present. A wireless keyboard is included with the system and slides into a shelf compartment at the bottom of the apparatus, but the ApeTouch software can also be fully controlled using a touch input on the human-facing control screen.

Apex measures 22 inches (width) by 16.5 inches (height) by 8.5 inches (depth) and is composed of a stainless-steel faceplate, a plexiglass assembly that houses the primate touchscreen (ELO 1590L Secure Touch with a screen area of 12 inches in width by 9 inches in height), and a polycarbonate shell that encases a “core” electronics box. The core box contains a pellet dispenser (Med Associates ENV203-190), a tablet PC (Microsoft Surface), a rechargeable and removable battery (Bix Power CP300), a digital input/output board (Phidgets 1012_2B), a 12 V power manifold, and a 12–24 V step-up converter for the pellet dispenser. The core box is hinged and swings out for easy access to swap the battery or refill the pellet hopper (Figure 1c). Apex secures to the mesh with two steel hooks at the top of the machine and two locking J-hook latch-clamps, which are adjustable along the vertical axis to fit a variety of different sizes of cage mesh. The hook and latch-clamp system allows for staff to securely put up or take down the machine in a matter of seconds, including whilst primates are present on the other side of the cage mesh. An LED light array surrounds the acrylic touchscreen assembly in the interior of the polycarbonate shell, which allows for the primate-facing side of the machine to glow green, red, or blue as feedback to primates during the touchscreen tasks. The rechargeable 300 watt/hour battery lasts for six hours of continuous use and can be quickly and easily swapped out for a second fully charged battery to maintain the running of the machine.

### 2.2. ApeTouch Software

**Figure 2:**
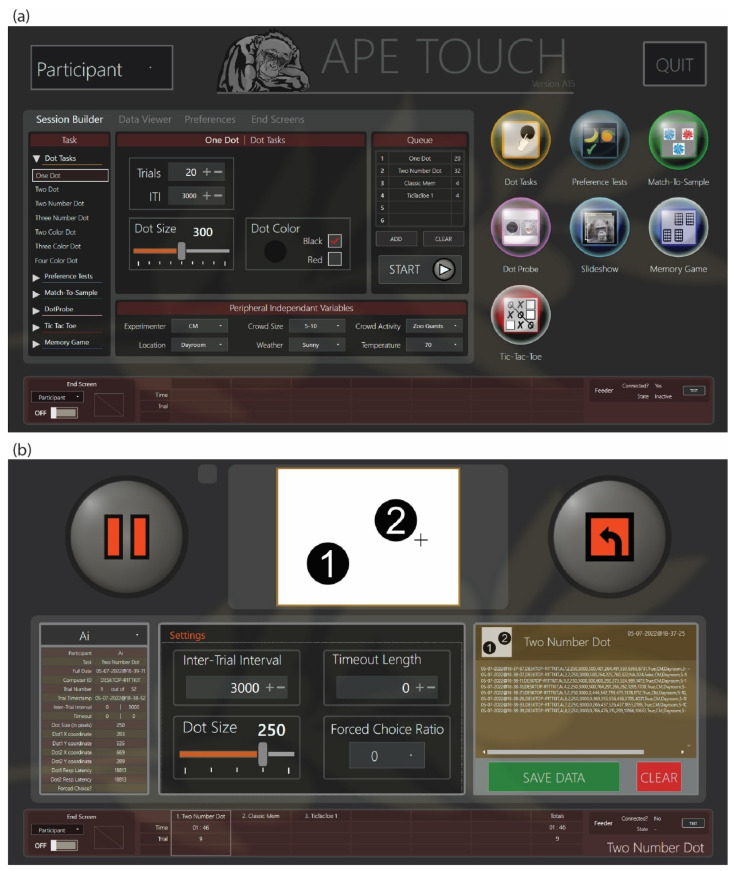
Screenshots of the ApeTouch user interface. (**a**) The main menu screen. On the right side are icons that lead directly to the control screen for each task. On the left are settings, preferences, and data panels as well as the session builder for creating session queues consisting of multiple tasks. (**b**) The control screen for the Two Number Dot task.

ApeTouch software is a Windows desktop application that runs on the Apex machine or on comparable PC/touchscreen systems. It consists of a variety of task modules (Figure 2a), including training exercises (Touch-The-Dot tasks), preference tests (choices between user-uploaded images), research tasks (Match-To-Sample, Dot Probe, Serial Ordering), and enrichment games (Memory Card game, Tic Tac Toe, Slideshow). Further details on these tasks are given in Supplementary Document S1. A simple and intuitive user interface along with streamlined navigation allows for human operators to quickly activate the tasks individually or set up a session queue consisting of multiple tasks. For each task, data on how the primate is using the task (timestamps, stimuli variables, response latency, performance) are automatically recorded and can be saved by the user for a later analysis. Many of the tasks allow the user to upload their own images, sounds, and trial sequence inputs or they may use the preset stimuli and built-in combinatorial engine, which automatically generates complete trial sequences. A key feature of the ApeTouch program is the dual-screen functionality; there is one screen for the human facilitating the task and another for the primate. The screen for the human user displays the navigation, setup, control, and task-monitoring information, including a picture-in-picture of what is being shown on the screen of the primate (Figure 2b). The screen of the primate only displays the contents of the tasks. From the main menu on the human screen, zoo staff can easily navigate and initiate a variety of tasks for their primates in a matter of seconds (as few as three touches to buttons on the control screen), making it very user-friendly (Figure 2a).

ApeTouch software has a track record of being used for published studies performed in zoo settings running on custom cart-based touchscreen systems or stationary wall-embedded touchscreen systems. These studies include examinations of serial ordering in gorillas and macaques [9,54]; preferences for food items in chimpanzees, gorillas, and macaques [30,45,55]; responses to an emotional Stroop task in gorillas, chimpanzees, and macaques [36]; macaque attentional bias caused by anthropogenic noise and zoo visitor attention [35,56]; performance on gambling tasks in chimpanzees, gorillas, and macaques [57]; attentional biases of apes toward familiar human faces [58]; orangutan visuospatial memory abilities [59]; and the effects of cognitive testing on macaque welfare [18].

The ApeTouch software framework and training tasks used in the current study are available for free in the Supplementary Materials (Software S1) and also as a free download on the Zenrichment website: Zenrichment.com (accessed on 26 June 2022). Ordering information for the full suite of ApeTouch tasks as well as the Apex portable touchscreen system can also be found on the Zenrichment website.

### 2.3. Subjects

Four group-housed macaque species at the Japan Monkey Centre in Inuyama, Japan, participated in the touchscreen training study (Table 1). None of the participating macaques had a prior history of touchscreen tasks except for one Bonnet macaque that had casually interacted with the smartphone of a keeper on several occasions. The sessions were conducted along a cage mesh wall of their outdoor enclosures. All groups had indoor–outdoor access to their living spaces and ad libitum access to water throughout the study. The study was approved by the ethics committee of the Japan Monkey Centre (Collaborative Research of Japan Monkey Centre: 2018019, 2019013, and 2020020) and conducted in accordance with the Japanese Act on the Welfare and Management of Animals and the Guidelines for Care and Use of Nonhuman Primates of Kyoto University Primate Research Institute.

### 2.4. Experimental Procedure

**Figure 3:**
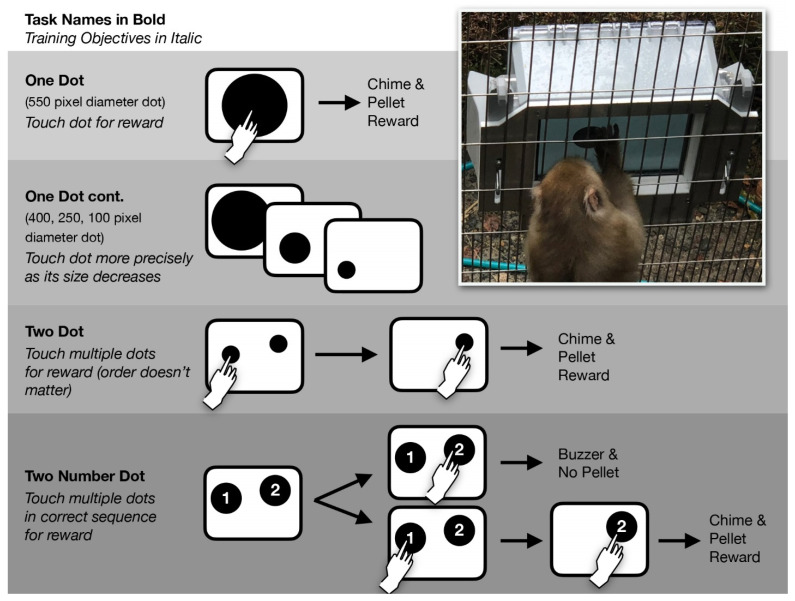
Graphical overview of the series of training tasks given to the four macaque groups. On the top right is a photo of a macaque completing the One Dot task.

The four groups of macaques were tested in their outdoor living spaces and were not moved or separated from their groupmates during the course of the study. The decision to conduct the study in a group setting rather than with individually separated primates was made because enrichment at zoos is typically applied to social settings [60]. The enclosures where the study took place were in public areas where zoo guests could observe the primates using the touchscreen (for a photo, see Supplementary Document S1). Prior to the start of the study, on a single occasion for each group, fruit jam was applied to the surface of the primate touchscreen to encourage investigation and interest in the apparatus. The study consisted of each group receiving one testing session per day over the course of 95 days, with sessions lasting for 10 min and having no set number of trials (subjects could complete as many trials as possible within the 10 min). The sessions began with the experimenter placing Apex on the cage mesh and initiating the ApeTouch tasks using the control interface on the human-facing screen (Figure 1 and Figure 2). The onset of the first trial of the session was accompanied by an initiation chime to alert the macaque group to the start of the session. The touchscreen tasks used in this study consisted of three tasks within the ApeTouch “Dot Tasks” module. These shaping tasks were designed to cultivate learning and comprehension in a step-by-step manner (Figure 3). Video footage of the study is available in the Supplementary Materials (Video S1).

The first task, named “One Dot”, consisted of a single black target dot presented at a random location against a white background. Touching the dot resulted in the disappearance of the dot on the screen, a chime sound, green LED illumination of the translucent touchscreen bezel, and the delivery of a banana-flavored grain pellet (Bio-Serv DPP 190 mg). This was followed by a brief inter-trial delay before the next trial began. For the first three sessions, the dot had a 550 pixel diameter (almost as large as the size of the screen), followed by two sessions with a 400 pixel diameter, two sessions with a 200 pixel diameter, and finally three sessions with a 100 pixel diameter. The training objectives for this task were to touch the stimulus and, as the dot decreased in size, to touch it precisely.

The second task, named “Two Dot”, consisted of two non-overlapping dots appearing at random locations on the screen at the onset of a trial. When a dot was touched, it disappeared. Once both dots were touched and had disappeared, the chime sounded and the subject was rewarded. Each group completed five sessions. The training objective for this task was to touch multiple stimuli.

The third task, named “Two Number Dot”, consisted of two dots appearing at random locations with a white Arabic numeral “1” on one of the dots and the numeral ”2” on the other. The aim of the task was to touch the dots in sequential order. If the “1” dot was touched first, it disappeared; when the remaining “2” dot was subsequently touched, the subject was rewarded. Alternatively, if the “2” dot was touched before the “1” dot, a buzzer sounded, red LEDs illuminated the touchscreen bezel, and no food pellet was delivered. A total of 80 sessions were carried out with each group. For the final 55 sessions, “forced choice” trials were introduced to facilitate learning. In these trials, which comprised 25% of the total trials given, only the “1” dot appeared. The overall training objectives for this task were to discriminate between multiple stimuli and to touch them in a specific order.

## 3. Results

### 3.1. Engagement

**Figure 4:**
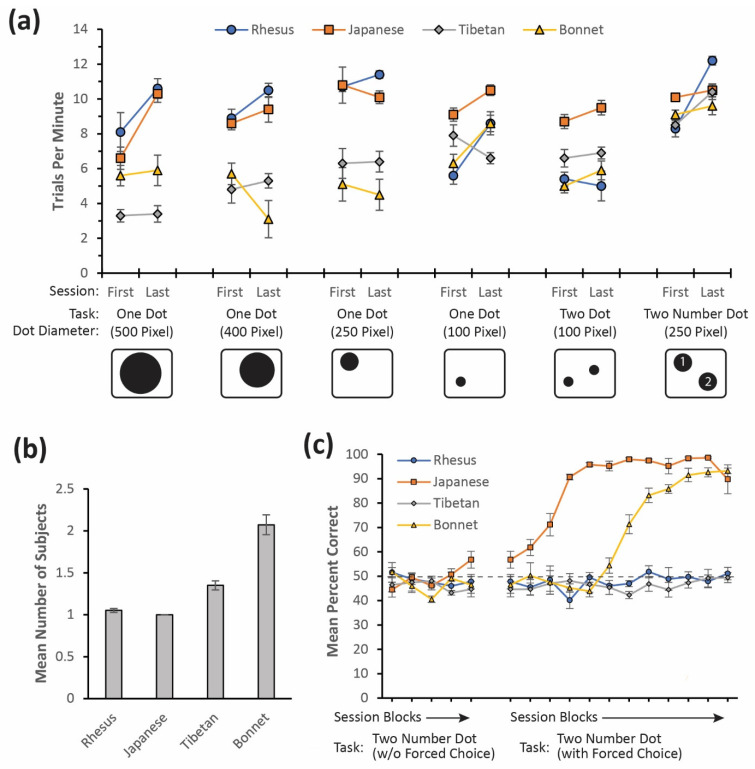
(**a**) Mean trials per minute for each group for the first and last session of each task. (**b**) Mean number of subjects that touched the screen for all sessions and tasks. (**c**) Performance rates on the Two Number Dot sequencing task. Blocks shown on the *x*-axis are composed of the five sessions each. Error bars show the mean standard error.

Engagement with the touchscreen was quantified using the mean number of trials completed per minute during the task sessions (Figure 4a), which served as a proxy for task-directed attention and behavior. Factoring in the inter-trial interval and the time requisite to find and touch the dot, the highest possible rate of usage was around 15 trials per minute. Figure 4a shows the trials per minute for each of the groups on each of the tasks. The usage rates began at a positive rate from the first exposure onward and generally increased between the first and last sessions of each task as well as over the course of the study as a whole. This was indicative of the continual and consistent usage of the device when it was present on the walls of the enclosures.

The mean number of individuals for each macaque group engaging in the touchscreen tasks across all sessions was manually recorded by the experimenter and is shown in Figure 4b. For two of the groups, Rhesus and Japanese, the device was monopolized by the dominant individual in the group whereas in the other two groups, Tibetan and Bonnet, the usage of the device was commonly shared among multiple members of the group.

### 3.2. Performance

The study included a sequencing task for which there was a correct and incorrect response; namely, the Two Number Dot task. Figure 4c shows the performance of each group over time with each data point representing a block of five sessions. The Two Number Dot task was divided into two phases. The first phase did not include any forced choice trials, but the second phase included them to bolster learning. The forced choice trials, which always resulted in a correct response, were removed from the performance analysis. The performance rates are shown in Figure 4c. The Japanese macaque group performed higher than the chance level starting on the fifth block for the Two Number Dot task without any forced trials mixed in (binomial test, *p* = 0.003); the Bonnet macaque group performed above the chance level starting on the sixth block with the forced choice trials mixed in (binomial test, *p* = 0.000). After exceeding the chance level, both these groups maintained a higher than chance performance for the duration of the study window. Neither the Rhesus nor the Tibetan macaque groups achieved an above-chance level performance during the study window, suggesting that continued training sessions were necessary for those groups.

## 4. Discussion

Our findings demonstrated a consistent engagement with the Apex touchscreen and ApeTouch software among all four groups of macaques over the course of the study, which amounted to roughly 16 h for each group spread across 95 days. As enrichment, the touchscreen captured the attention and behavior of at least one member of each group on every session of the study, and in the case of the Bonnet macaques, several group members jointly participated. Interestingly, the two macaque species in which a single individual monopolized the touchscreen, Rhesus and Japanese, have been characterized in prior studies as having despotic dominance styles whereas the other two species that showed higher rates of joint participation, Bonnet and Tibetan, have been characterized as egalitarian [61]. This finding suggests that the utilization of touchscreens by macaque groups in zoo settings follows the manner of their natural species-typical dominance styles [62].

The subordinate individuals in each group who did not use the touchscreen may have benefitted from the presence of the touchscreen in incidental ways. As pointed out in a prior enrichment work by Markowitz [23], when dominant individuals monopolize an enrichment device, the subordinate individuals are often temporarily freed of the watchful gaze and potential agonism of the dominant. However, it may also have been the case that the non-participating subordinate individuals experienced increased stress levels from seeing food being dispensed, but not being able to access it due to the monopolization of the dominant. One possibility for remedying the inability of subordinates to directly gain access to the touchscreen when a dominant is present is to give the device to subordinates when they are individually housed in holding areas. Another possibility is to provide additional enrichment opportunities that would distract the dominant individual, thereby freeing up the touchscreen for use by subordinates. Yet another possibility is to simply offer multiple devices spread apart from each other. This tactic has been successfully employed with a chimpanzee group at Kyoto University Primate Research Institute where multiple separate “walk-in booths” with touchscreen stations are situated along the walls of outdoor enclosures [63]. Likewise, Fagot and colleagues have designed a system of ten networked touchscreen stations for baboons that are accessible from an outdoor enclosure space [64].

The training regimen outlined in this study followed a step-by-step process for cultivating basic touchscreen skills and comprehension. The task objectives advanced from touching the screen to touching the stimuli precisely, then from touching multiple stimuli to touching the stimuli in a particular sequence. All four macaque groups completed the first three objectives; two of the four groups completed all of them. Crucially, the last objective introduced the notion of a “correct” and “incorrect” way of responding, which is a prerequisite for more elaborate enrichment games and research tasks, including other ApeTouch tasks such as Match-To-Sample and the Memory Card game. Two groups in the current study, the Japanese macaques and the Bonnet macaques, achieved a better than chance performance on the sequencing task, putting them in a position to move on to those and other more complex tasks. The other two groups, Rhesus and Tibetan, actively engaged in the tasks, but did not exceed the chance level in the study window. Future possibilities for increasing their performance rate might include the addition of more forced choice trials or the introduction of a time-out delay after incorrect responses, which are both features of the training software (Figure 2b).

The design of the Apex machine and ApeTouch software, including the dot training tasks utilized for the current study, were largely influenced by our prior work on the Ai Project of the Kyoto University Primate Research Institute. For over four decades, the Ai Project has examined the perceptual and cognitive abilities of a multi-generational group of chimpanzees. Since its beginning in 1977, the central feature of the project has been voluntary cognitive research sessions utilizing automated methods, including a wide variety of touchscreen tasks [6,7,65]. Elements of the Ai Project touchscreen methodology that are incorporated into ApeTouch tasks include step-by-step training, discrete trials with start keys, sound and food reinforcement as well as a general principle of “stimuli on the screen should always be touchable”, overall task simplicity to promote comprehension and motivation, and an emphasis on objectivity, precision, and detailed record-keeping [7].

Another key aspect of the Ai Project that we sought to replicate in the zoo setting of the current study using Apex/ApeTouch was the creation of more naturalistic feeding patterns. The touchscreen sessions gave the macaque groups an opportunity to intermittently feed over an extended period of time based on their own physical and mental effort. This is a more naturalistic scenario than typical handfeeding at zoos and it invites opportunities for contrafreeloading, a phenomenon whereby animals choose to work and earn food rather than take it freely from feeding troughs or dishes [66,67]. In the current study, the sessions lasted for 10 min, but future applications for the Apex machine and ApeTouch software could involve longer sessions to serve as a prolonged enrichment for alleviating boredom. Future efforts with these macaque groups could also involve adjusting the timing and frequency of the touchscreen sessions to coincide with wild macaque temporal foraging patterns. Along these lines, the chimpanzees of the Ai Project are normally given four opportunities per day to participate in the computer sessions, which, combined with their three meals, amounts to seven distinct feeding bouts. This number was informed by the observations of wild chimpanzees in Bossou, Guinea, that averaged seven feeding bouts per day. Yamanashi and Hayashi [68] compared daily activity budgets between the Ai Project chimpanzees and the wild chimpanzees in Bossou and found that access to touchscreen sessions resulted in a comparable allotment of daily time spent feeding and travelling between the two groups. Such considerations for utilizing technology to promote naturalistic activity budgets offer another potential benefit for the use of touchscreens at zoos.

## 5. Conclusions

The development of Apex and ApeTouch relied on a foundation of long-established and proven methodologies for primate touchscreen work in laboratory settings whilst simultaneously considering the unique challenges and opportunities of zoo settings. The portability of the machine allows it to be easily transported and hung on the enclosures of multiple species, as we have demonstrated with a study involving four macaque species in different enclosures at JMC Zoo. For zoo staff, the human-facing screen combined with the intuitive user interface of ApeTouch makes it easy to operate and control. For primates, the step-by-step training process, variety of mentally stimulating tasks, and automatic dispensing of food results in continual engagement and interest. For all of these reasons, the Apex portable touchscreen system and ApeTouch software represent promising steps forward for the adoption of technology-based primate enrichment and scientific efforts at zoos.

## Figures and Tables

**Table 1:** The four macaque groups that participated in the training study.

Species	Number of Individuals
Rhesus macaque (*Macaca mulatta*)	4
Japanese macaque (*Macaca fuscata*)	5
Tibetan macaque (*Macaca thibetana*)	5
Bonnet macaque (*Macaca radiata*)	15

## Data Availability

The datasets that we analyzed for the current study are available from the corresponding author on reasonable request.

## Supplementary Materials

The following supporting information can be downloaded at: https://www.mdpi.com/article/10.3390/ani12131660/s1, Video S1: Footage of macaque study and Apex apparatus; Document S1: Overview of ApeTouch touchscreen tasks; Software S1: Zip folder containing installation files for the ApeTouch Dot tasks used in this study.
