# Supplementary material for: Apex and ApeTouch: Development of a Portable Touchscreen System and Software for Primates at Zoos
Source: Animals (Basel). 2022 Jun 28;12(13):1660. doi: 10.3390/ani12131660 (PMC9265006; doi:10.3390/ani12131660)
Supplement: Supplementary file 1 [file animals-12-01660-s001.zip › Document_S1.pdf]

Supplemental Online Material

Apex and ApeTouch: Development of a Portable Touchscreen System and Software for Primates at Zoos

Christopher Flynn Martin, Akiho Muramatsu, Tetsuro Matsuzawa

Contents

A. Overview of ApeTouch Software Touchscreen Tasks

I. Training Tasks

- Dot Tasks Page 2

II. Research Tasks

- Match-to-Sample Page 3
- Dot Probe Page 4

III. Husbandry Aides

- Preference Tests Page 5

IV. Enrichment Games

- Memory Card Game Page 6
- Tic-Tac-Toe Page 7
- Slideshow Page 8

B. Photograph of guests observing JMC Zoo study

I. Figure S1 Page 9

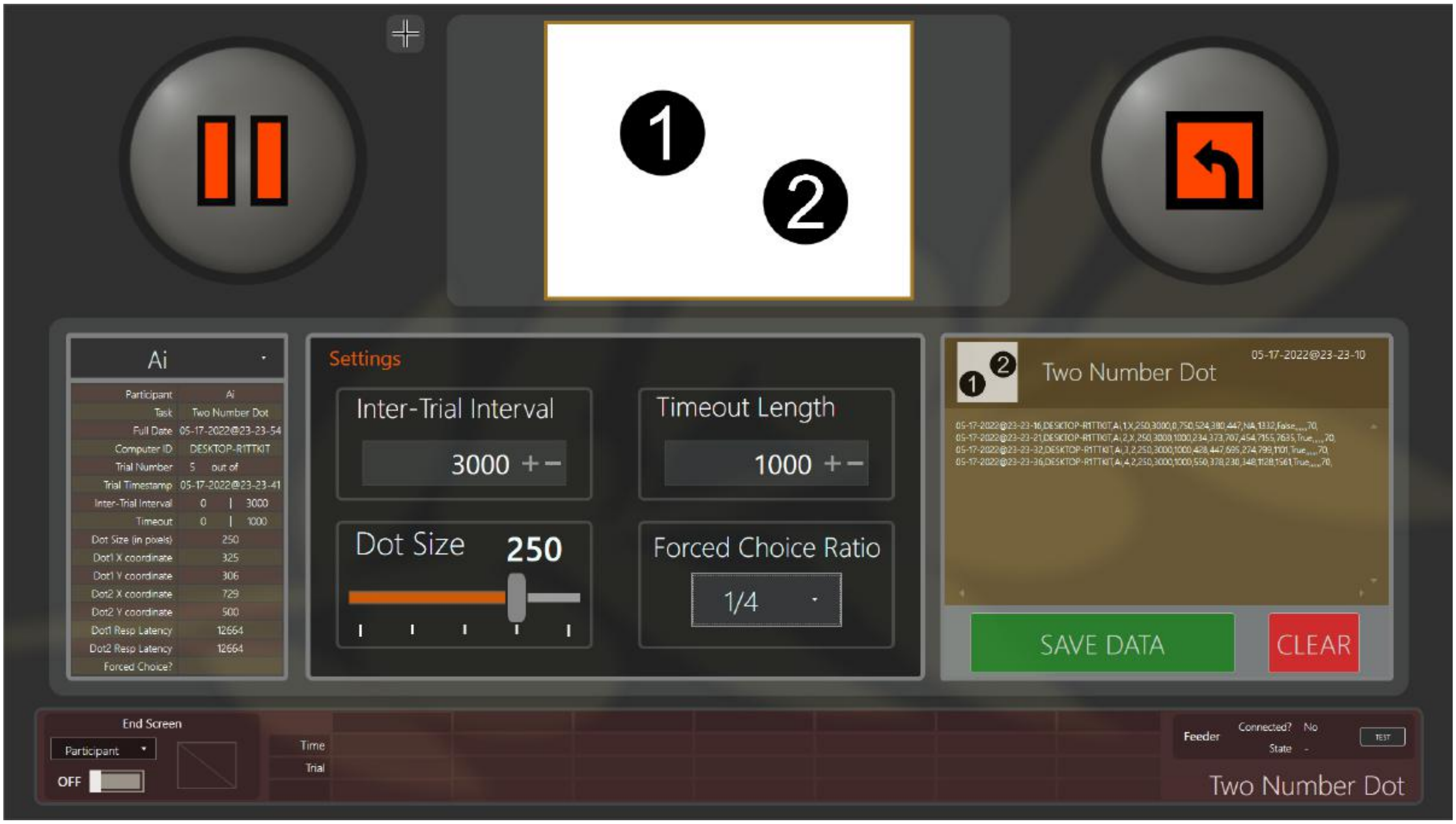

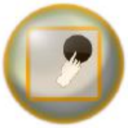

Dot Tasks

Features:

- Multiple touch-the-dot tasks for step-by-step training of touchscreen-inexperienced primates
- Operator can control the size of the dot(s) and inter-trial interval.

One Dot

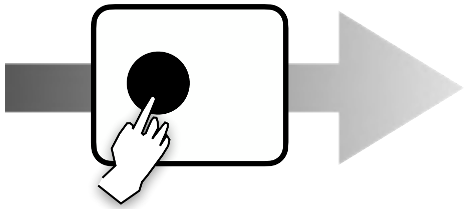

Two Dot

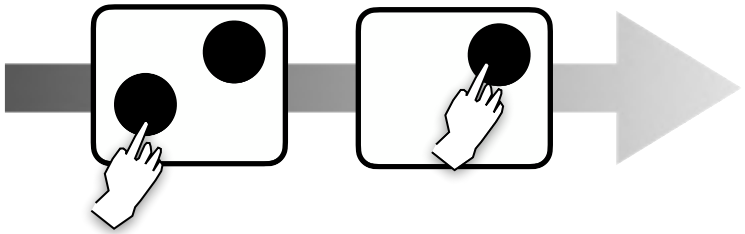

Two Number Dot

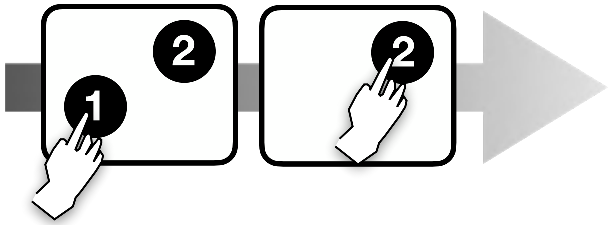

Three Number Dot

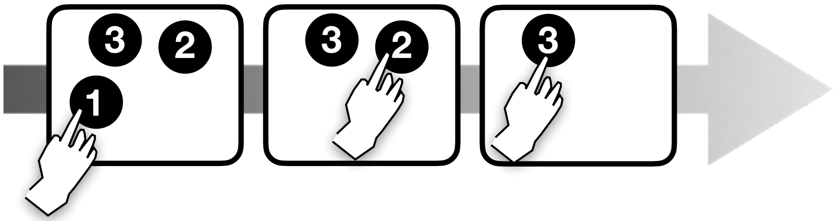

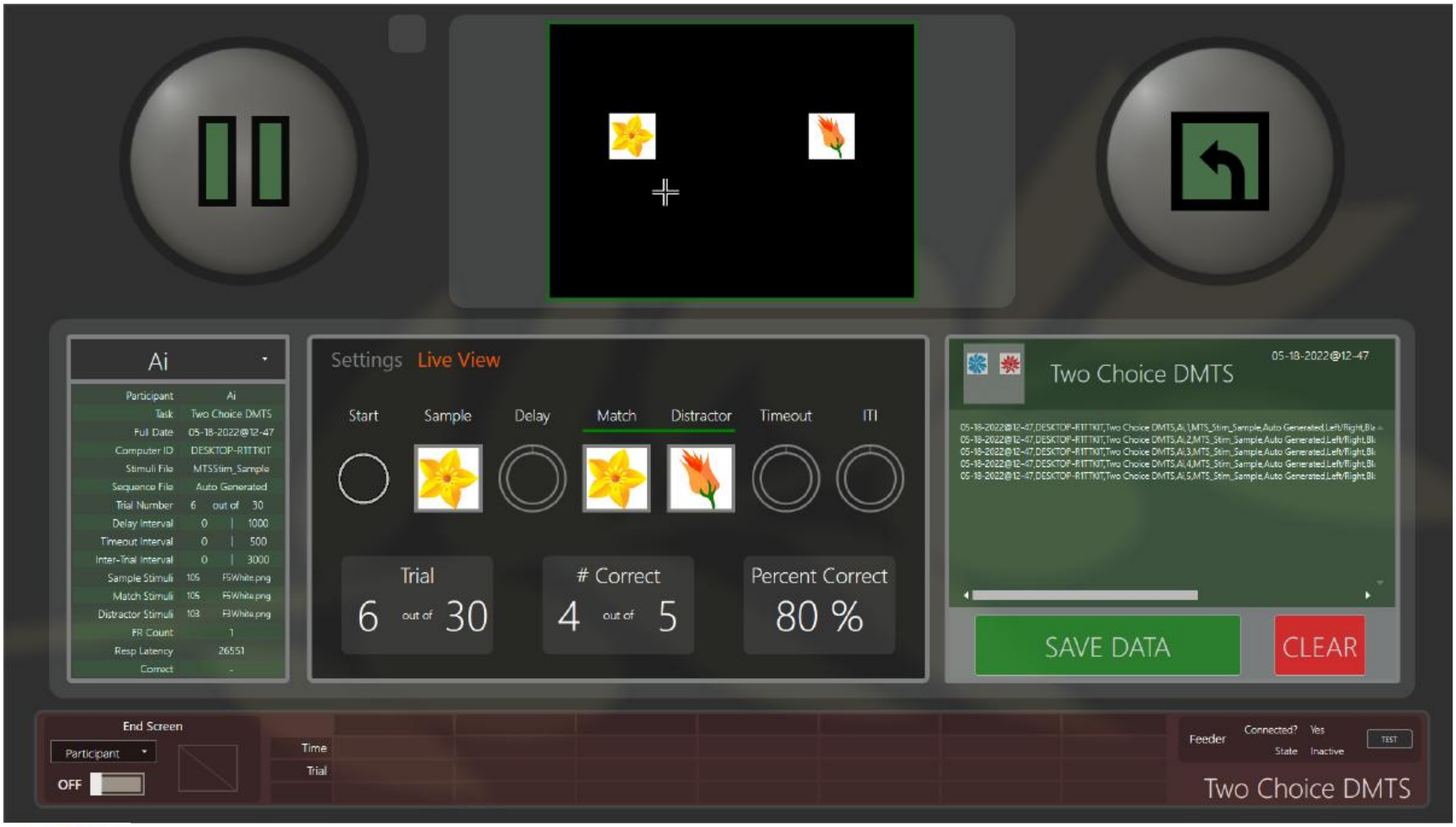

Match-To-Sample

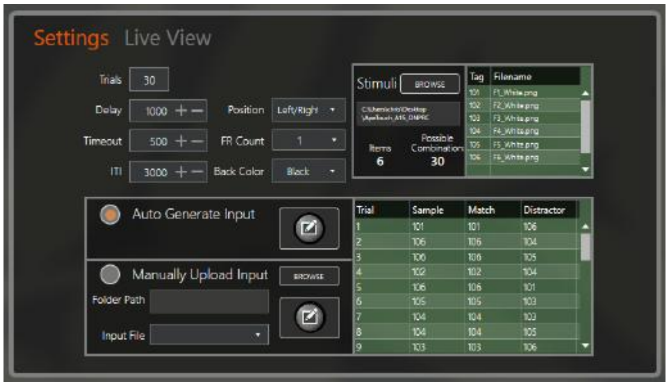

Features:

- A classic psychology task paradigm.
- Operator can upload their own image sets.
- Operator can upload a custom trial sequence file or use the Auto Generate function.
- Parameter controls include:
  Delay, Stimuli Position, Timeout, Sample FR Count, Inter-trial Interval, Background Color

<u>Simultaneous Match-To-Sample</u>

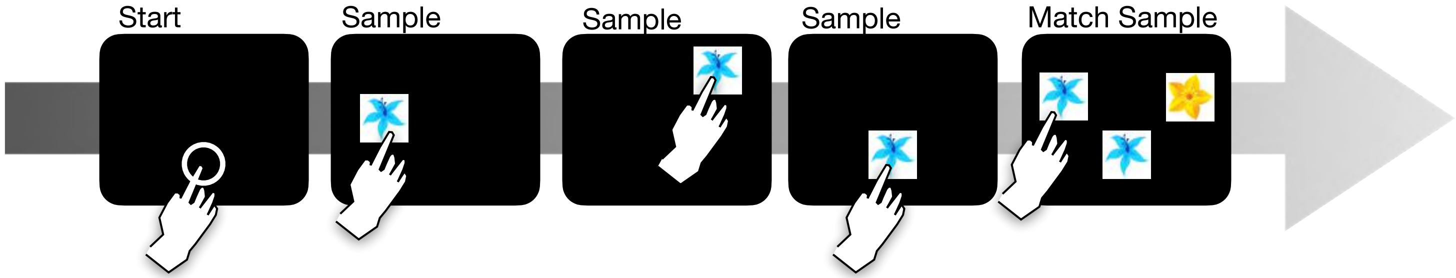

<u>Delayed Match-To-Sample</u>

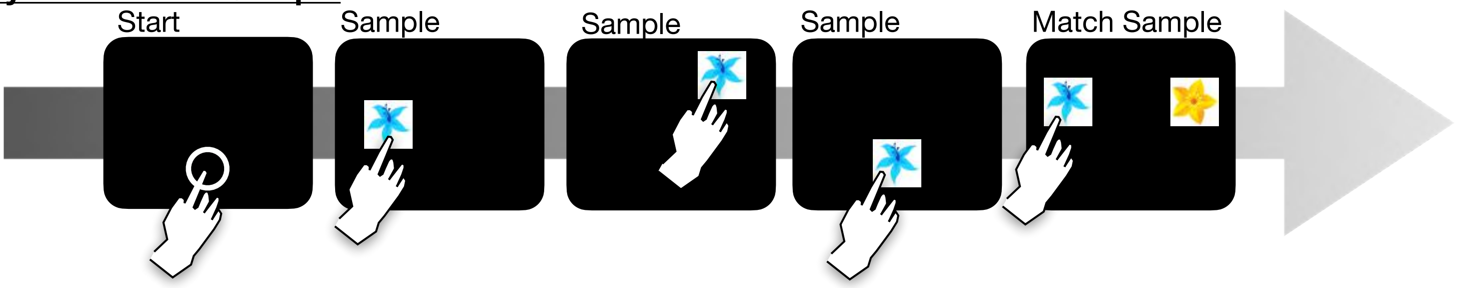

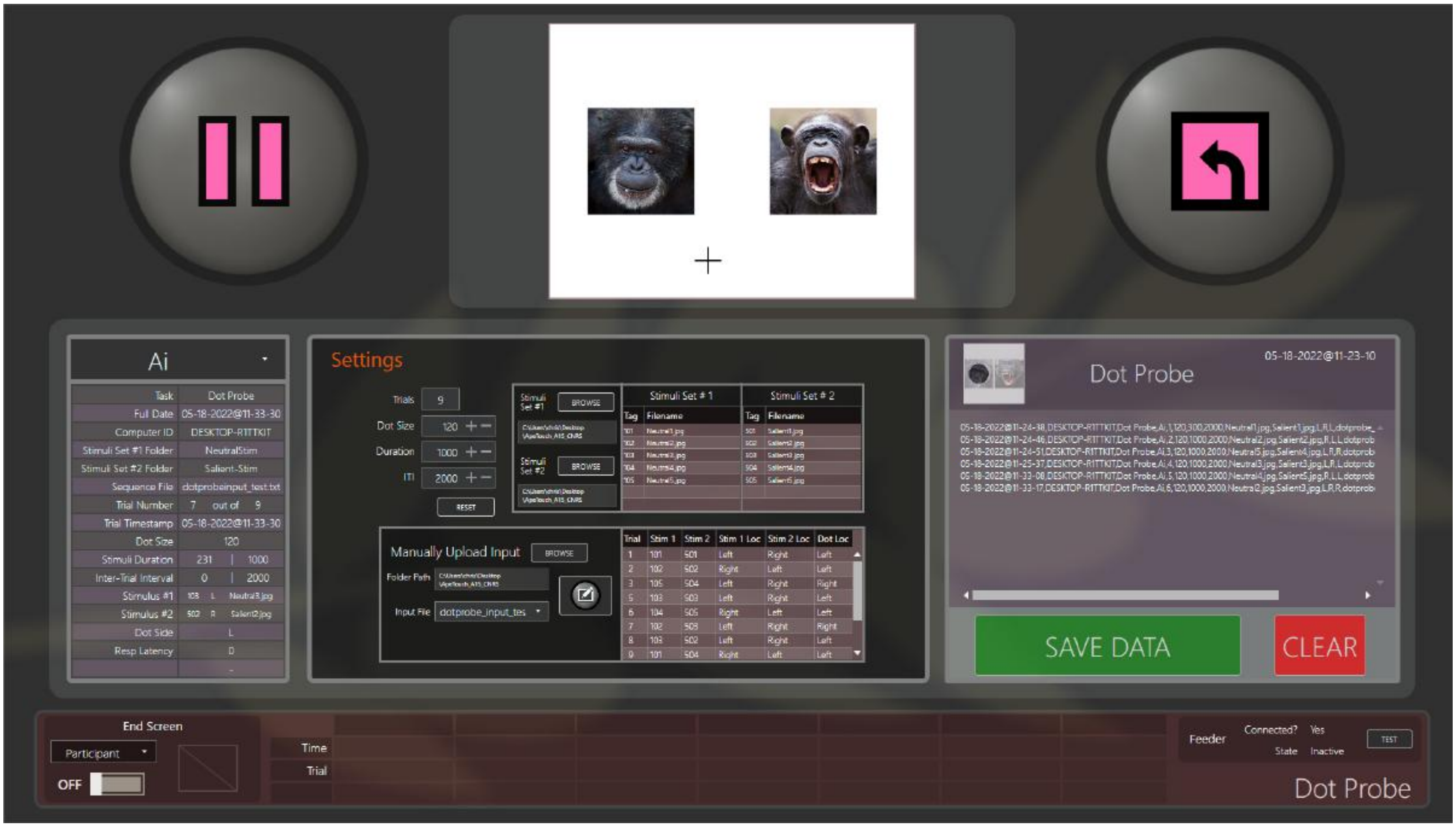

Dot Probe

Features:

- Popular psychology task paradigm for testing attentional bias toward different kinds of stimuli.
- Operator can upload their own image sets and trial sequences.
- Parameter controls include:

    Dot size, Stimuli Onset Duration, Inter-trial interval

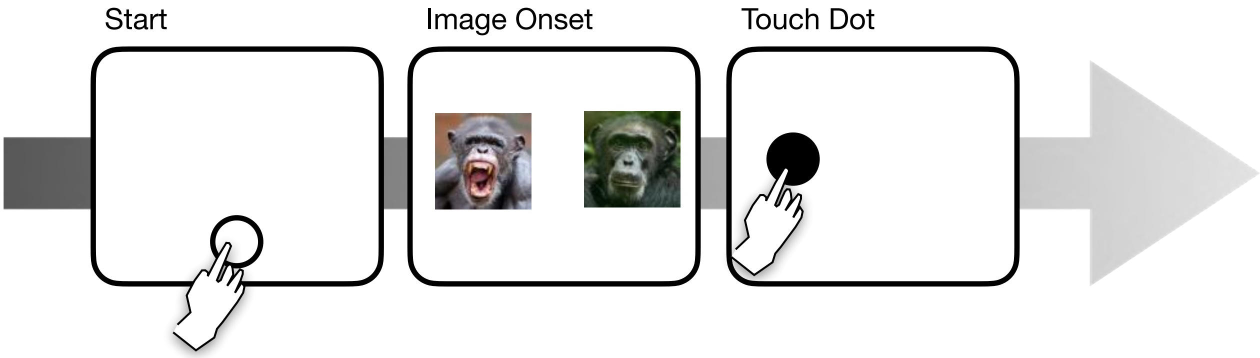

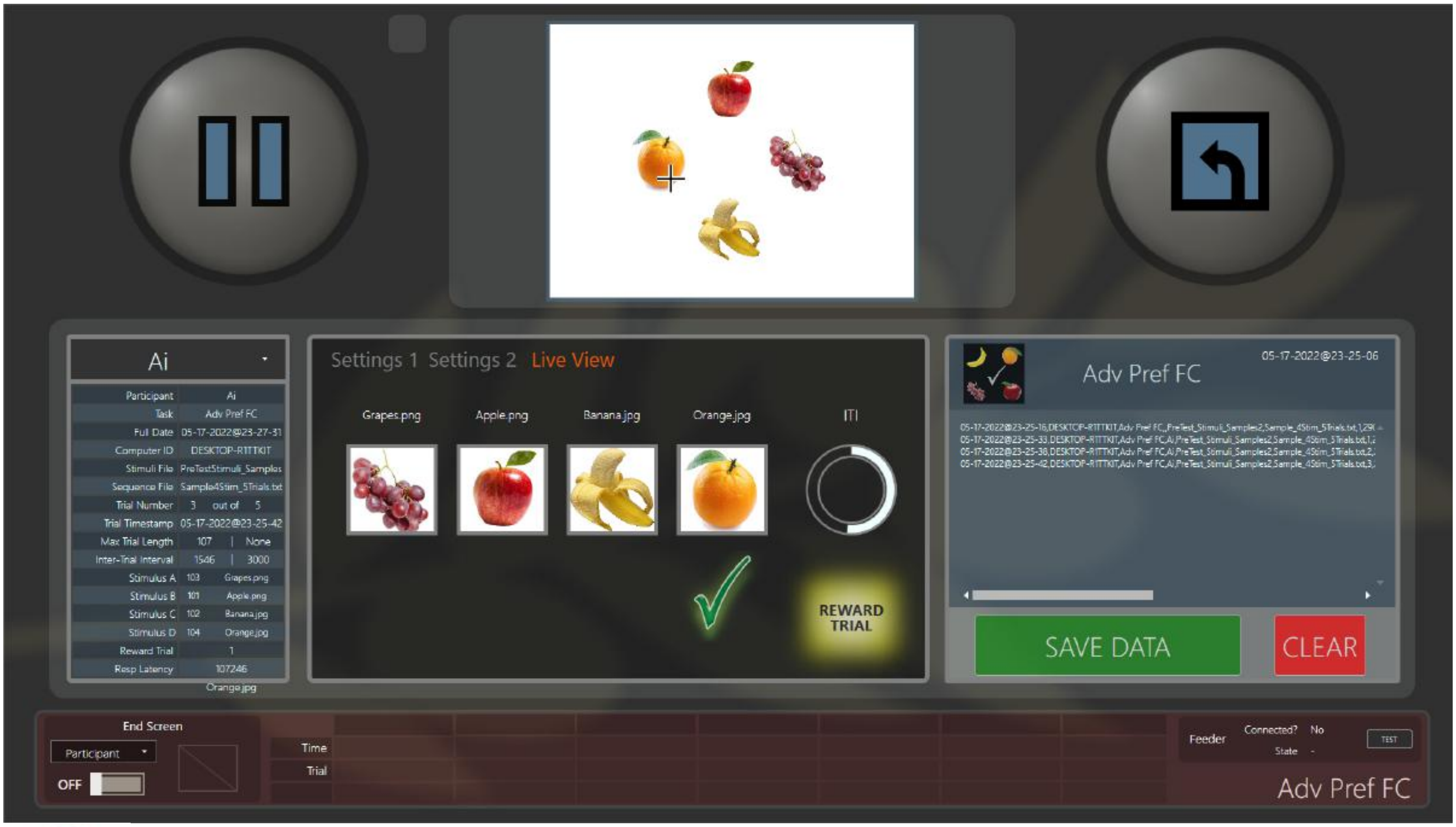

Preference Tests

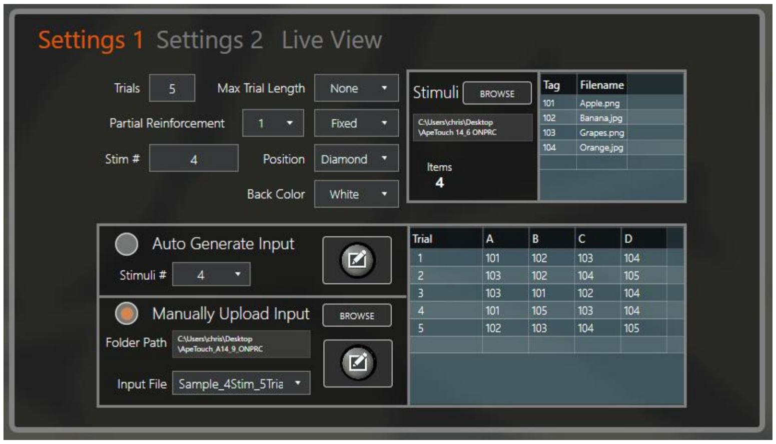

Features:

- Present images for primates to choose from (for example, foods, locations, faces).
- Operator can upload their own image sets.
- Operator can upload a custom trial sequence file or use the Auto Generate function.
- Parameter controls include:

  Stimuli number per trial, Stimuli positions on the screen, Background color, Maximum trial length, Inter-trial interval, Reinforcement sounds

Images shown Choose image

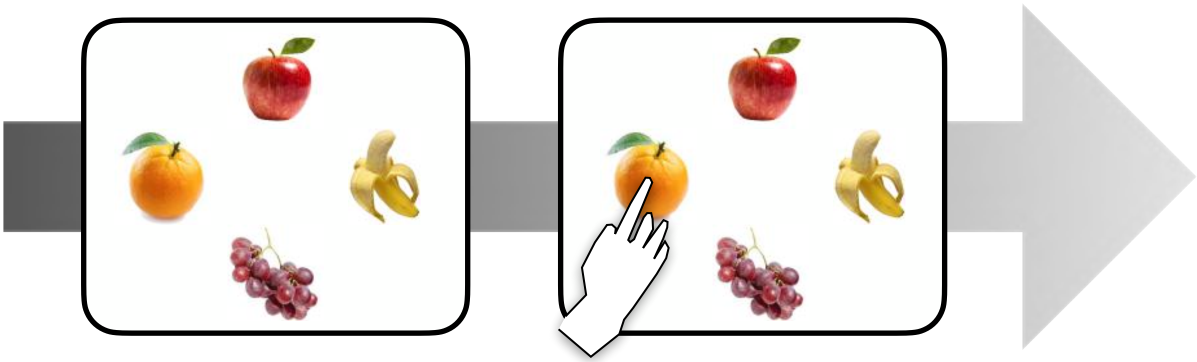

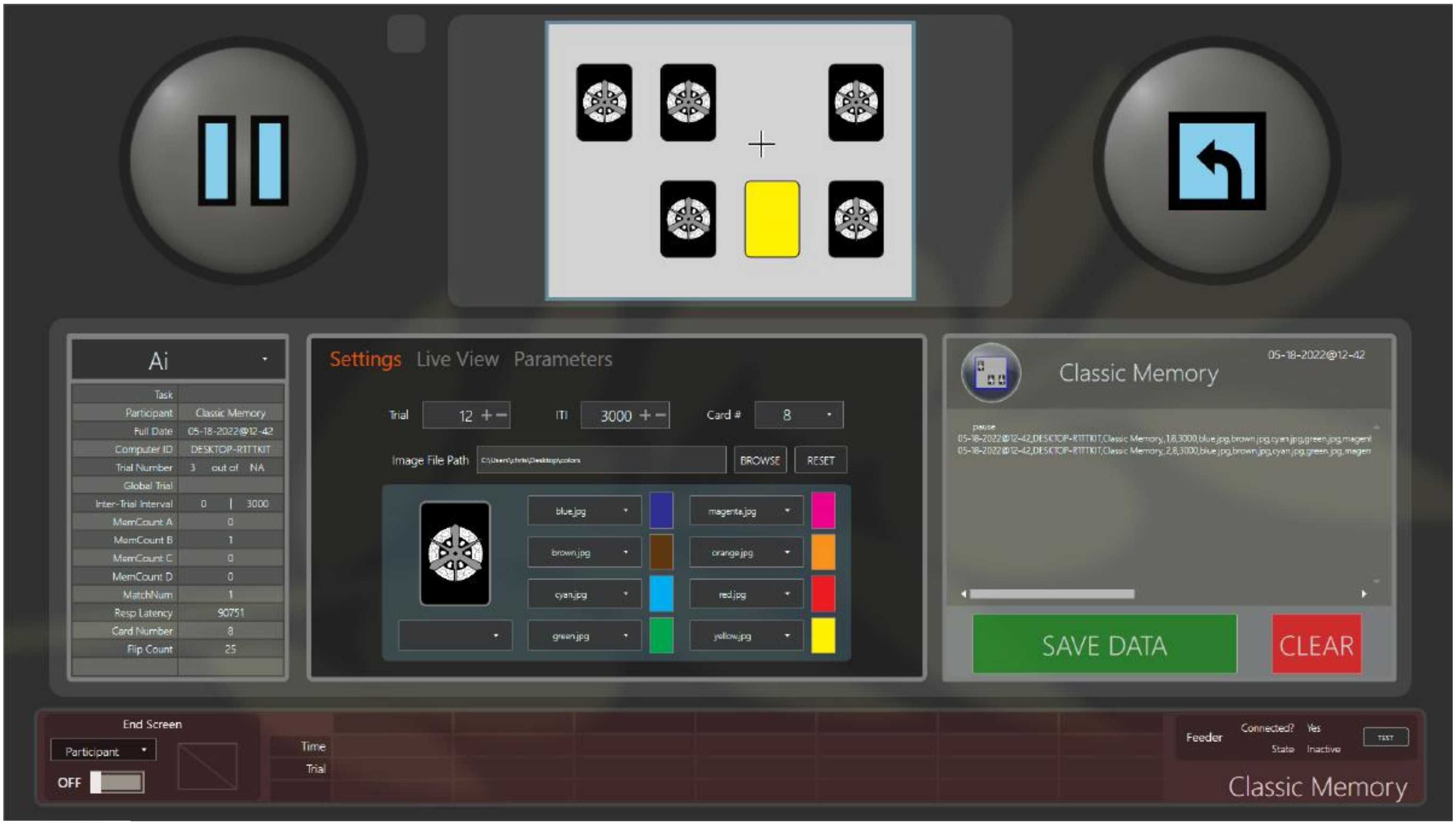

Memory Game

Features:

- Classic children's card game which also serves as a psychology test for visuospatial memory [see: Martin, C & Shumaker, R (2022) Orangutan strategies for solving a visuospatial memory task. *American Journal of Primatology*, e23367].
- Operator can upload custom card images.
- Parameter controls include:
  Number of card pairs presented, inter-trial interval

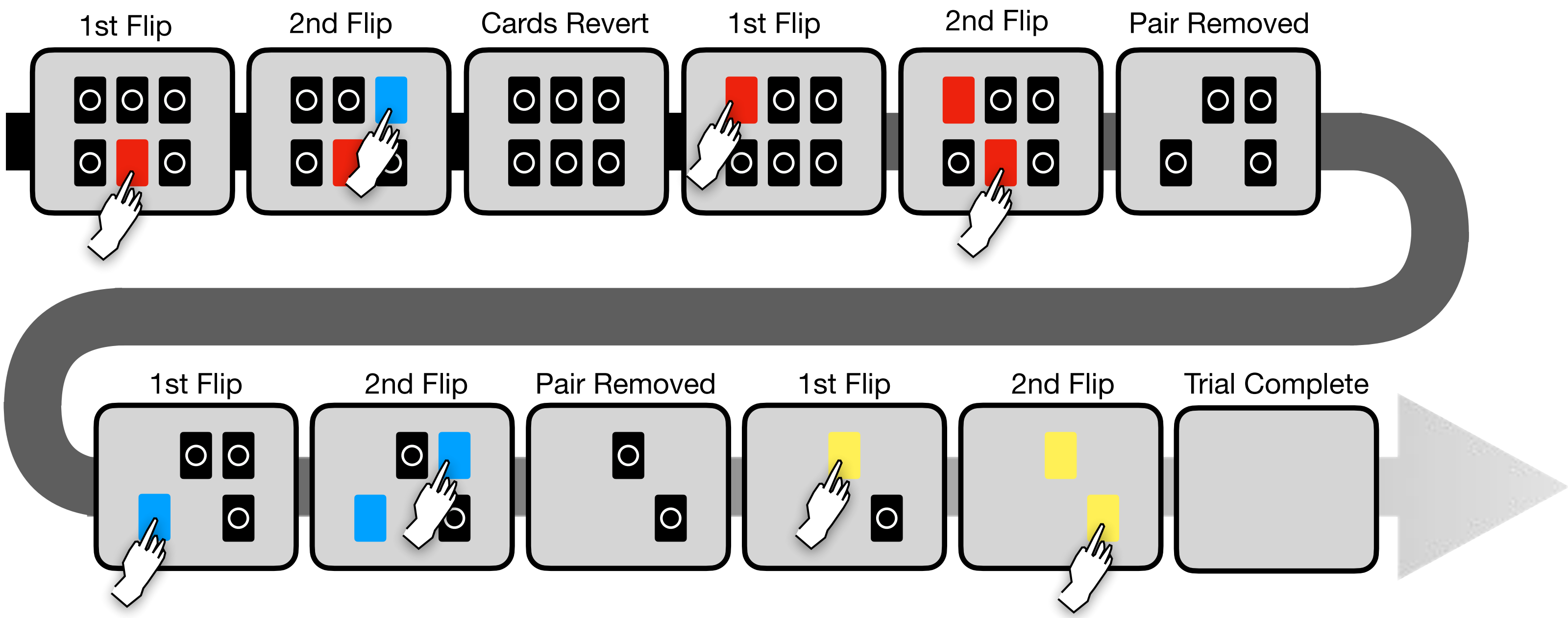

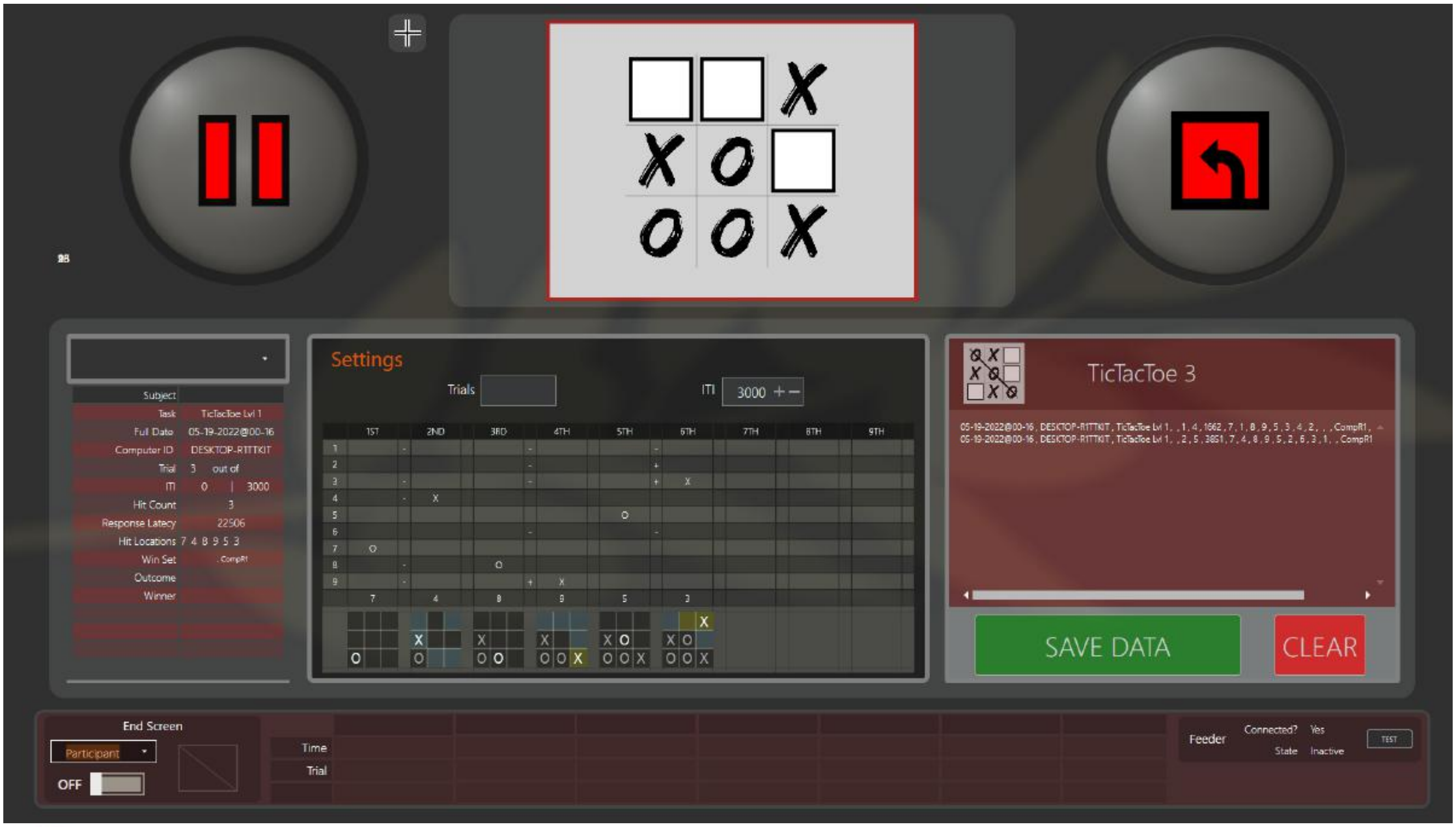

Tic-Tac-Toe

Features:

- Classic game of tic-tac-toe.
- A dynamic algorithm plays in the opponent-role with three levels of difficulty.
- The algorithm prevents primates from winning the same way every time.

Trial where primate wins

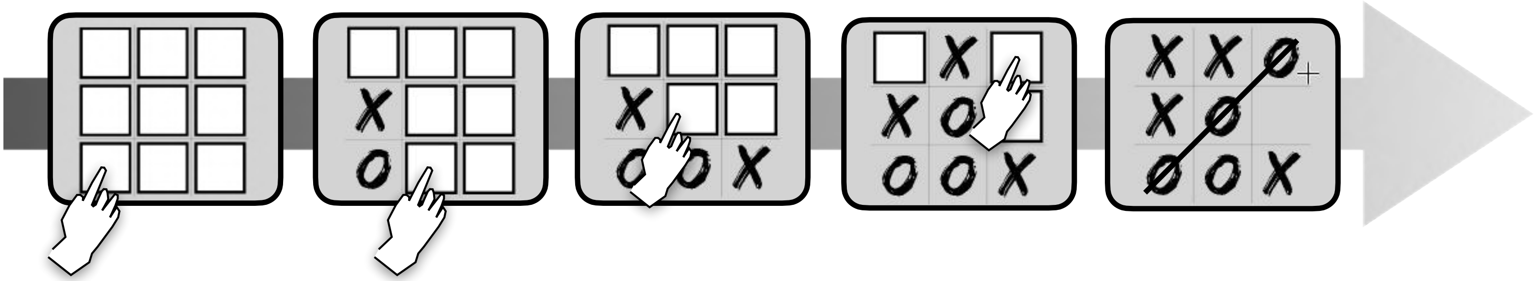

Trial where computer wins

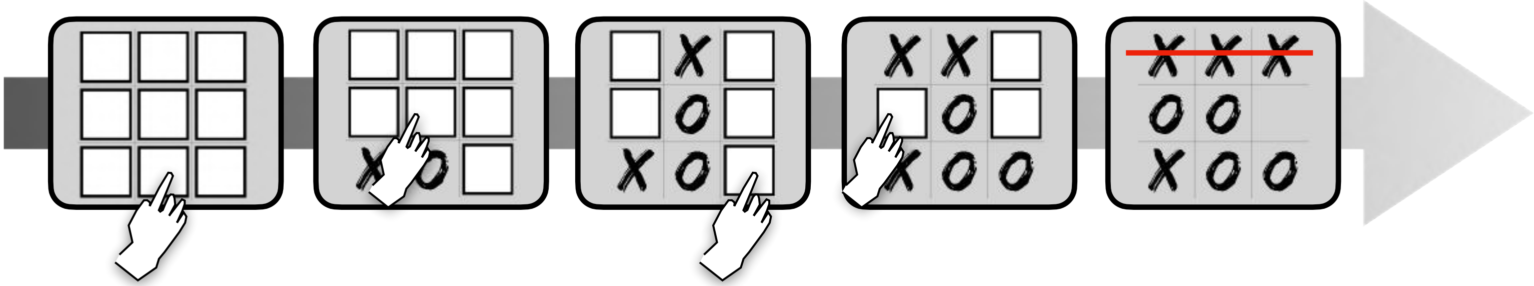

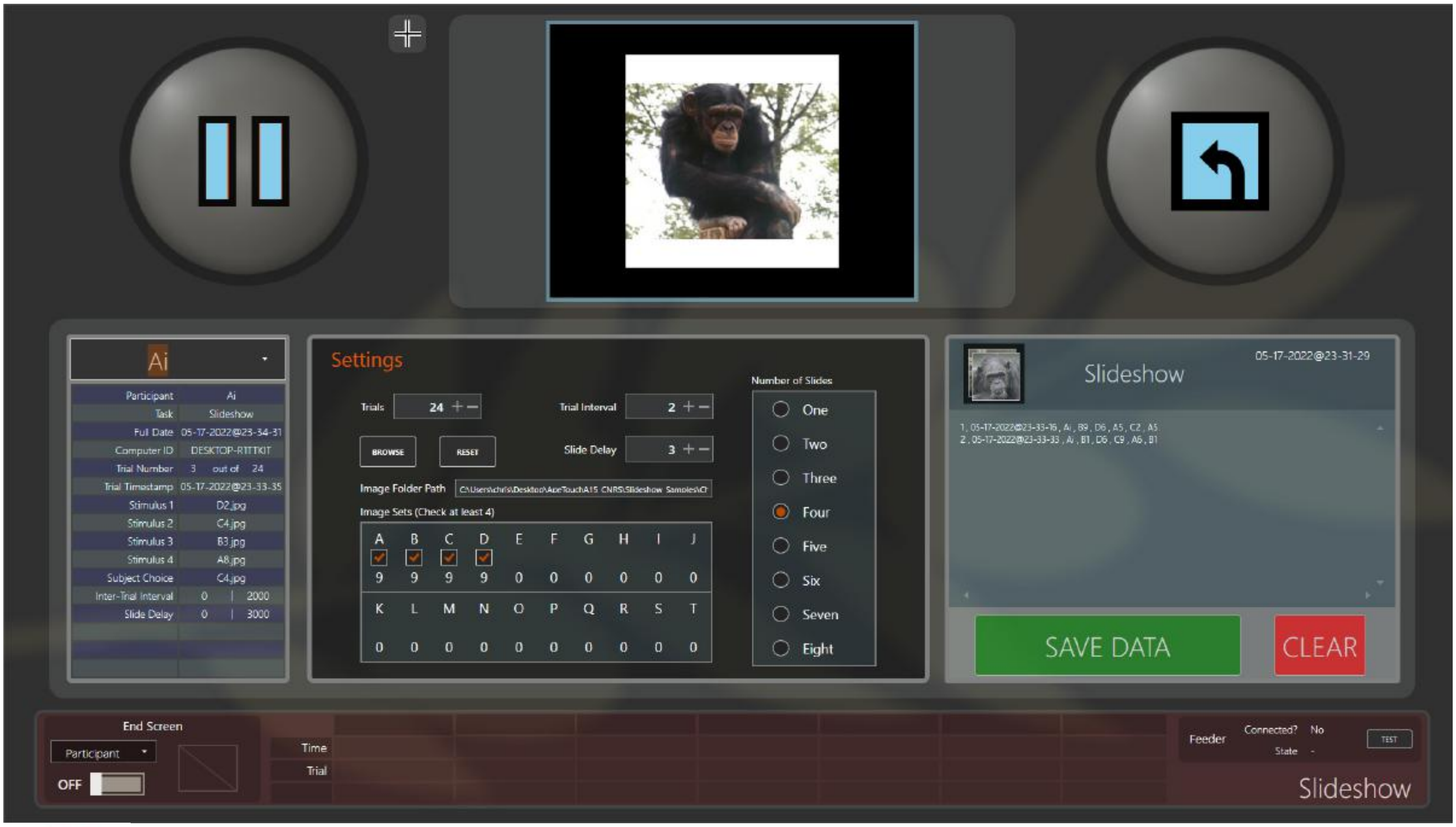

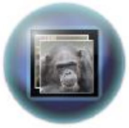

Slideshow

Features:

- Enrichment activity that lets primates choose and view photo albums.
- Operator uploads image sets that comprise each album.
- Parameter controls include:

  Inter-trial interval, delay between slides, number of slides per trial

Choose image Album of slides of the chosen image is presented

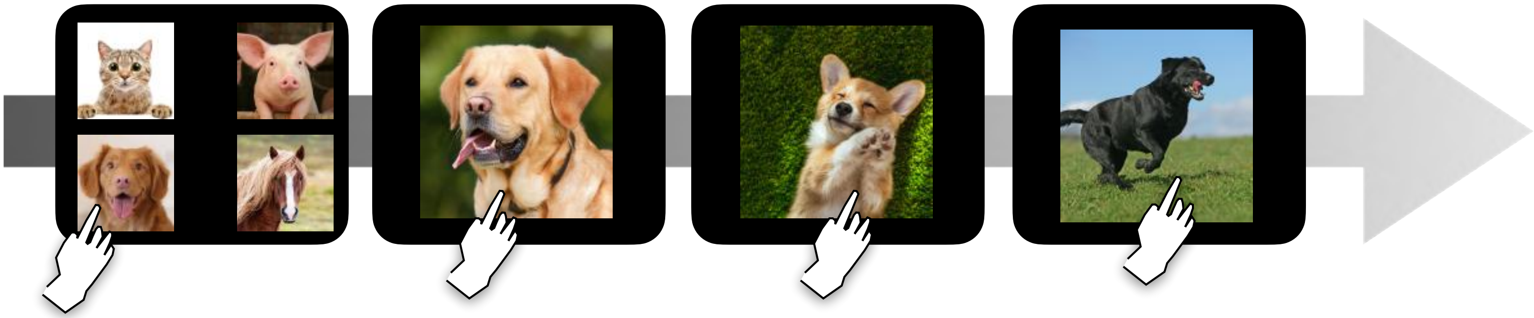

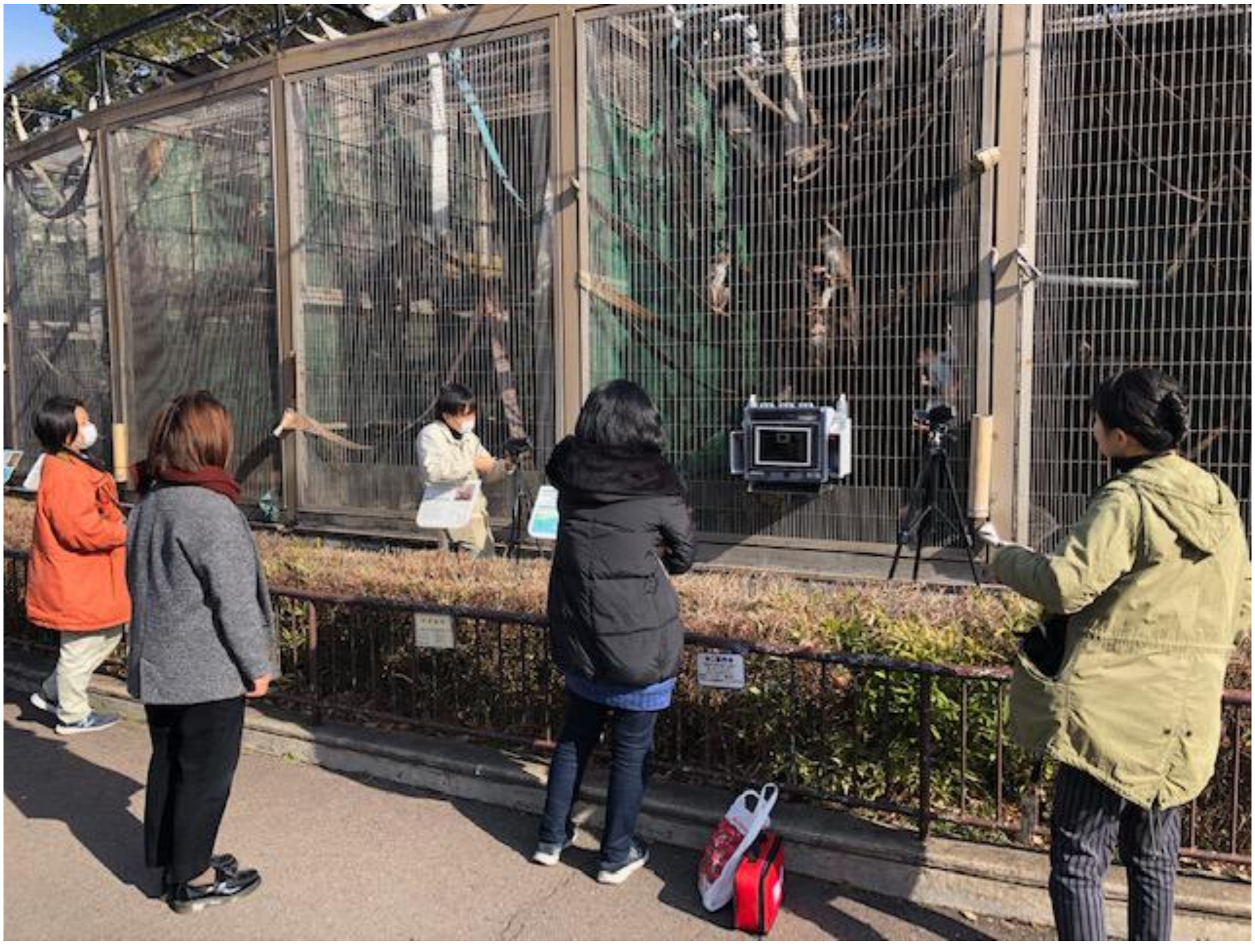

Figure S1. View of a macaque enclosure from the visitor area at JMC Zoo. Guests observe the training study taking place involving the Apex apparatus and ApeTouch software.
